# Supplementary figures and images for: Exploring the impact of health literacy on pregnant women from ethnic minority groups: A scoping review
Source: PLoS One. 2024 Dec 31;19(12):e0312515. doi: 10.1371/journal.pone.0312515 (PMC11687770; doi:10.1371/journal.pone.0312515)

Appendix 1: PRISMA checklist


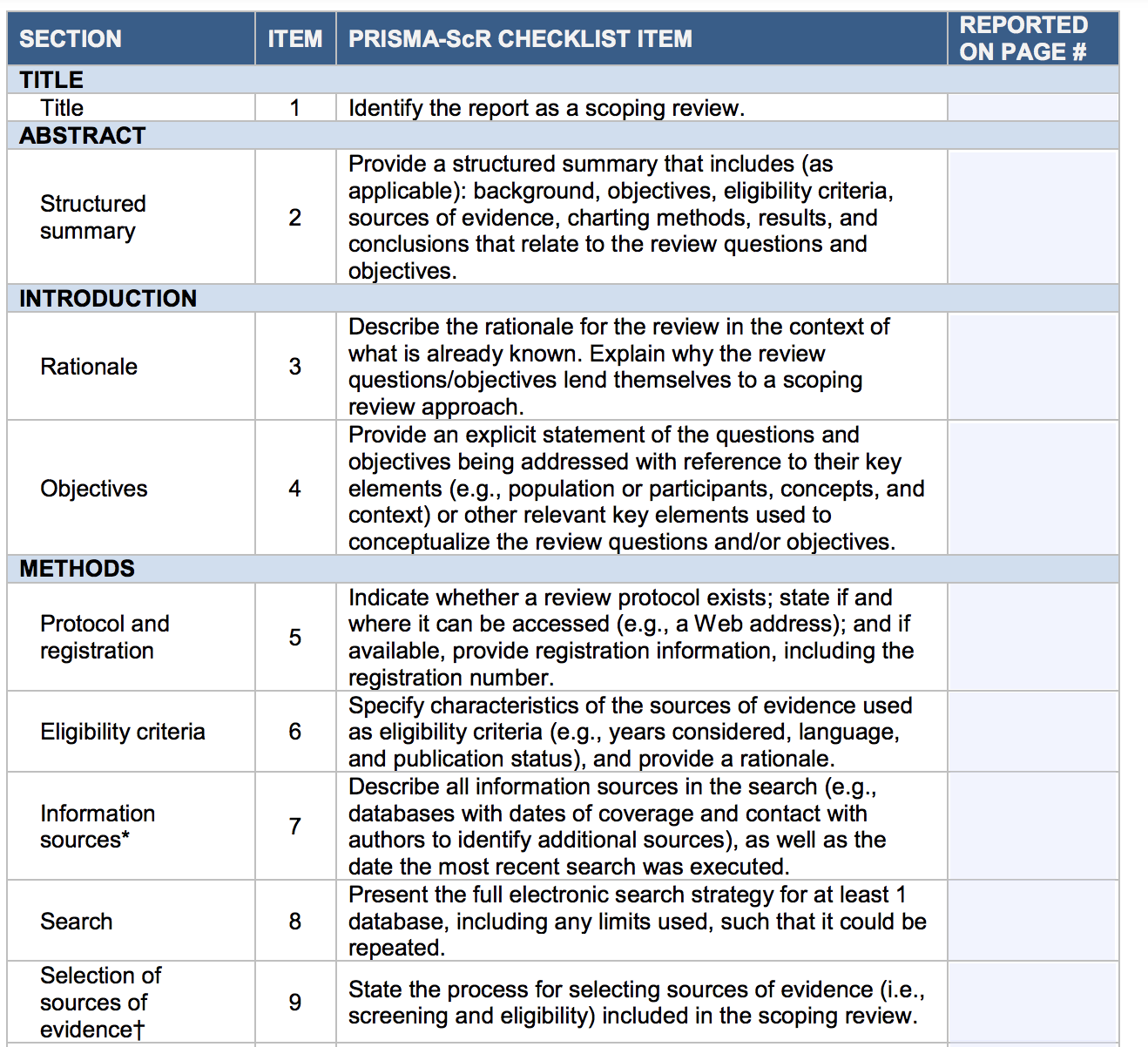


7

29-30

6

5-6

4-5

4

3-4

2

1


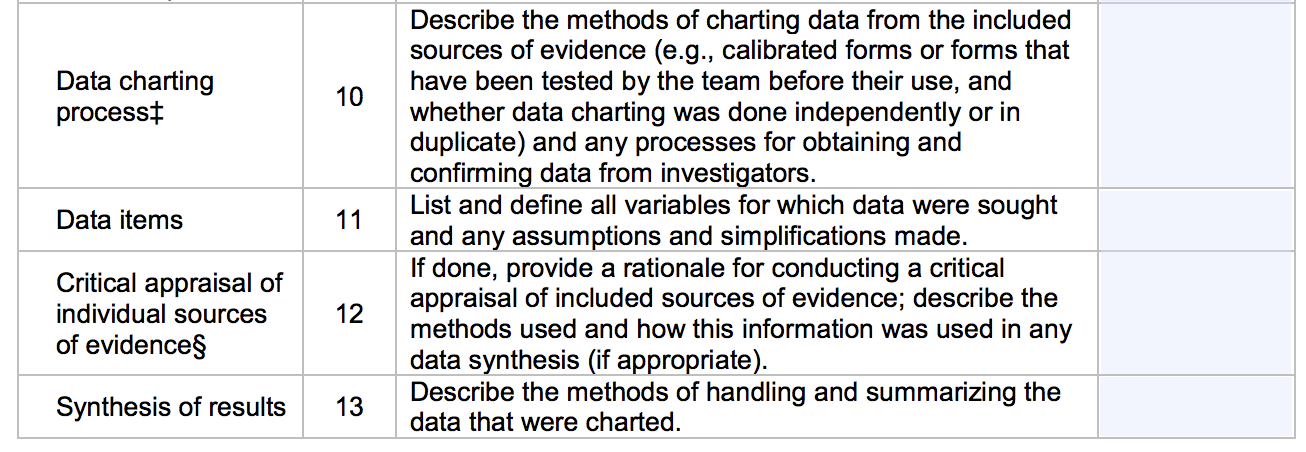


7

N/A

6

9


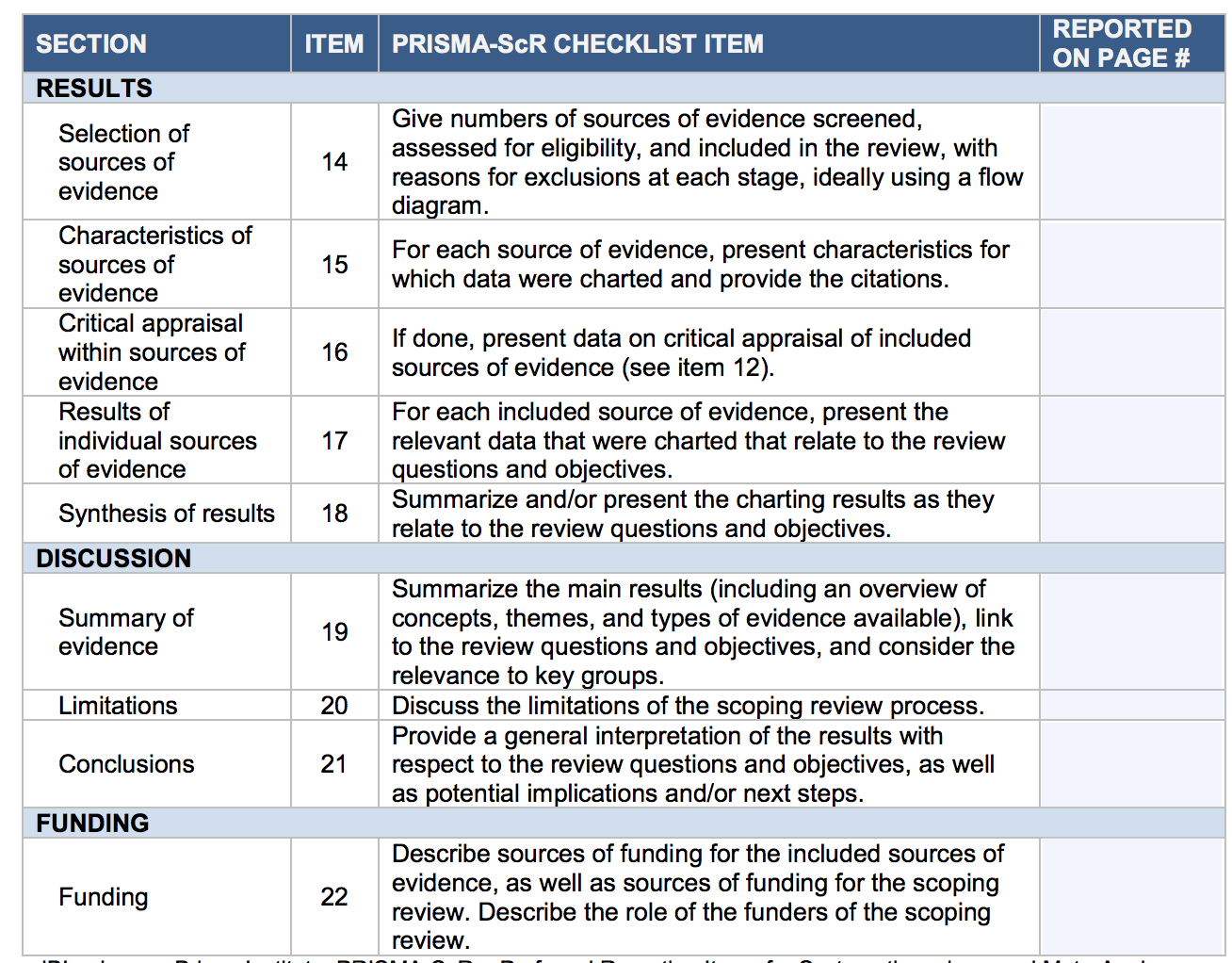


22-23

21

11-16

19-23

11-19

10-11

1

N/A

7-8

Supplement: S1 Checklist — (DOCX) [file pone.0312515.s001.docx]
