# Supplementary material for: Exploring the impact of health literacy on pregnant women from ethnic minority groups: A scoping review
Source: PLoS One. 2024 Dec 31;19(12):e0312515. doi: 10.1371/journal.pone.0312515 (PMC11687770; doi:10.1371/journal.pone.0312515)
Supplement: S1 Appendix — (DOCX) [file pone.0312515.s003.docx]

S3- Appendix : Search Strategy

ALL <1946 to June 29, 2023>, Maternity & Infant Care Database (MIDIRS) <1971 to

June 27, 2023>

Search Strategy:

1pregnan*.mp. [mp=ti, ab, hw, tn, ot, dm, mf, dv, kf, fx, dq, bt, nm, ox, px, rx, ui, sy,

ux, mx] (2537478)

2antenatal.mp. [mp=ti, ab, hw, tn, ot, dm, mf, dv, kf, fx, dq, bt, nm, ox, px, rx, ui, sy,

ux, mx] (137002)

3prenatal.mp. [mp=ti, ab, hw, tn, ot, dm, mf, dv, kf, fx, dq, bt, nm, ox, px, rx, ui, sy,

ux, mx] (525682)

4gestation.mp. [mp=ti, ab, hw, tn, ot, dm, mf, dv, kf, fx, dq, bt, nm, ox, px, rx, ui, sy,

ux, mx] (370280)

5childbirth.mp. [mp=ti, ab, hw, tn, ot, dm, mf, dv, kf, fx, dq, bt, nm, ox, px, rx, ui, sy,

ux, mx] (91005)

6maternal health.mp. [mp=ti, ab, hw, tn, ot, dm, mf, dv, kf, fx, dq, bt, nm, ox, px, rx,

ui, sy, ux, mx] (55256)

7maternal welfare.mp. [mp=ti, ab, hw, tn, ot, dm, mf, dv, kf, fx, dq, bt, nm, ox, px,

rx, ui, sy, ux, mx] (25026)

81 or 2 or 3 or 4 or 5 or 6 or 7 (2851920)

9BAME.mp. [mp=ti, ab, hw, tn, ot, dm, mf, dv, kf, fx, dq, bt, nm, ox, px, rx, ui, sy, ux,

mx] (1034)

10BME.mp. [mp=ti, ab, hw, tn, ot, dm, mf, dv, kf, fx, dq, bt, nm, ox, px, rx, ui, sy, ux,

mx] (6014)

11ethnic minority.mp. [mp=ti, ab, hw, tn, ot, dm, mf, dv, kf, fx, dq, bt, nm, ox, px, rx,

ui, sy, ux, mx] (22497)

12(ethnic or racial group).mp. [mp=ti, ab, hw, tn, ot, dm, mf, dv, kf, fx, dq, bt, nm, ox,

px, rx, ui, sy, ux, mx] (338673)

13(black or African or Caribbean or Asian or Indian or Pakistani or Somali or

Bangladeshi).mp. [mp=ti, ab, hw, tn, ot, dm, mf, dv, kf, fx, dq, bt, nm, ox, px, rx, ui, sy,

ux, mx] (1462045)

14(mixed or mixed race).mp. [mp=ti, ab, hw, tn, ot, dm, mf, dv, kf, fx, dq, bt, nm, ox,

px, rx, ui, sy, ux, mx] (1167010)

15non-english speaking.mp. [mp=ti, ab, hw, tn, ot, dm, mf, dv, kf, fx, dq, bt, nm, ox,

px, rx, ui, sy, ux, mx] (4452)

169 or 10 or 11 or 12 or 13 or 14 or 15 (2795088)

17health literacy.mp. [mp=ti, ab, hw, tn, ot, dm, mf, dv, kf, fx, dq, bt, nm, ox, px, rx,

ui, sy, ux, mx] (41855)

18health education.mp. [mp=ti, ab, hw, tn, ot, dm, mf, dv, kf, fx, dq, bt, nm, ox, px,

rx, ui, sy, ux, mx] (242222)

19patient education.mp. [mp=ti, ab, hw, tn, ot, dm, mf, dv, kf, fx, dq, bt, nm, ox, px,

rx, ui, sy, ux, mx] (244617)

20health understanding.mp. [mp=ti, ab, hw, tn, ot, dm, mf, dv, kf, fx, dq, bt, nm, ox,

px, rx, ui, sy, ux, mx] (1135)

21self-advocacy.mp. [mp=ti, ab, hw, tn, ot, dm, mf, dv, kf, fx, dq, bt, nm, ox, px, rx,

ui, sy, ux, mx] (1681)

22patient-advocacy.mp. [mp=ti, ab, hw, tn, ot, dm, mf, dv, kf, fx, dq, bt, nm, ox, px,

rx, ui, sy, ux, mx] (50692)

23health knowledge.mp. [mp=ti, ab, hw, tn, ot, dm, mf, dv, kf, fx, dq, bt, nm, ox, px,

rx, ui, sy, ux, mx] (135946)

2417 or 18 or 19 or 20 or 21 or 22 or 23 (667310)

258 and 16 and 24 (4885)

26remove duplicates from 25 (3987)
