# Supplementary material for: Exploring the impact of health literacy on pregnant women from ethnic minority groups: A scoping review
Source: PLoS One. 2024 Dec 31;19(12):e0312515. doi: 10.1371/journal.pone.0312515 (PMC11687770; doi:10.1371/journal.pone.0312515)
Supplement: Attachment — Submitted filename: Response to Reviewers.docx [file pone.0312515.s004.docx]

Dear Dr Julia Morgan,

Thank you for your consideration of the manuscript titled “Exploring the Impact of Health Literacy on Pregnant Women from ethnic minority groups A Scoping Review” - (PONE-D-24-25675). We highly value the time and effort spent by your office and your reviewers to propose suggestions to improve the above-mentioned manuscript. We present an itemised tabulated responses to the comments as per your request. We genuinely believe that by addressing the comments the manuscript has significantly improved.

We look forward to hearing from you. Please do not hesitate to contact us for any further queries.

Truly,

Dr Keivan Armani (on behalf of the authors)

| Journal Comments | Our Response |
| --- | --- |
| 1) With the patient representations did you use PCC or PEO or PICO to design the research question? | We used the PCC method to design our research question. This has been added to the methods. This is reflected on the page 4 and 5 of the revised manuscript as:  “We chose the Patient/Public, Concept, and Context (PCC) framework to formulate and finalise the research question. As our research relied on the lived experiences of patients/public, we found the PCC framework to be the most appropriate framework to properly acknowledge both the context of maternal health outcomes and the general concept of health literacy.” |
| 2) With your narrative analysis - did you use thematic analysis - how did you do your analysis of text? | The qualitative content analysis of the text in our manuscript was “descriptive” in nature. We did not apply content/thematic analysis techniques, as these techniques are not prescribed for scoping reviews. We acknowledge that JBI’s meta-aggressive methos were beyond the scope of the work.  This has been added to the methodology section on page 7. |
| 3) In the PRISMA flowchart - the word 'wrong' is used - can you use an alternative word for why excluded rather than wrong. | Thank you for highlighting this. We have updated the PRISMA flow chart on page 9.  We have removed the word “wrong”; and have replaced it with the following terms:  Different outcomes  Different indication  Incorrect study design  Incorrect patient population |
| Journal requirements |  |
| Please ensure that your manuscript meets PLOS ONE's style requirements, including those for file naming. The PLOS ONE style templates can be found at  <https://journals.plos.org/plosone/s/file?id=wjVg/PLOSOne_formatting_sample_main_body.pdf> and  <https://journals.plos.org/plosone/s/file?id=ba62/PLOSOne_formatting_sample_title_authors_affiliations.pdf> | Thank you for the reminder. We have ensured the journal specifications are conformed to. |
| Please include a copy of Table 3 which you refer to in your text on page 7. | Thank you for highlighting this error. We have corrected the label from Table 3 to Table 2, throughout the manuscript.  Table 2 has been added to page 11. |
| Please include captions for your Supporting Information files at the end of your manuscript, and update any in-text citations to match accordingly. Please see our Supporting Information guidelines for more information: <http://journals.plos.org/plosone/s/supporting-information>. | Thank you for highlighting. This has been amended and the appendixes have been updated to supplemental information with captions as seen on pages 31 and 32. |
| Please review your reference list to ensure that it is complete and correct. If you have cited papers that have been retracted, please include the rationale for doing so in the manuscript text, or remove these references and replace them with relevant current references. Any changes to the reference list should be mentioned in the rebuttal letter that accompanies your revised manuscript. If you need to cite a retracted article, indicate the article’s retracted status in the References list and also include a citation and full reference for the retraction notice. | Thank you for highlighting. References have been reviewed and reference 12, originally Lang et al, has been updated to Joseph et al, on reflection. The original reference was older evidence that has been updated with more up to date information that we found supported our data. |
| Reviewer 1 Comments | Our Response |
| Thank you for undergoing this critical research on "Exploring the Impact of Health Literacy on Pregnant Women from ethnic minority groups". This work highlights the need for more primary research that investigates the impact of health literacy on maternal outcomes and patient experience among these marginalised groups. Very well done to all the authors! | We appreciate your positive feedback. |
| The setting was not very clear to the reader.  Issue (I): The included articles and conclusion suggest a global focus while some sections of the introduction point towards the United Kingdom and the United States. For example, Page 3 paragraph 1 suggests a focus on the UK while Page 3 paragraph 3 suggests an focus on the US and the UK. This is not the case with Page 3 paragraph 3, where several articles from different countries have been included, majority of them been from Denmark and the US.  Suggestion (S): You may want to establish the setting more clearly and introduce data from more settings in the introduction rather than the UK or the US as they do not seem sufficient, considering the global focus. Alternatively, you may consider highlighting the lack of data from other places, if this is the case. | Thank you for pointing this out. We referred to your suggestion and have amended our description of the setting in the research and have made the scope of the setting more global.  This has been mentioned on page 3:  There have been a series of reports published on the increasing maternal mortality rates of ethnic minority women globally, as seen in the articles found within this scoping review (8-12, in addition to articles in table 2) Although the majority of the literature comes from Global North, there were a couple of studies from Global South (18,19,24), which on its own seems to be highly debatable and could highlight the lack of data in the peer-reviewed academic literature (15,16) |
| Conflicting date limitations in eligibility criteria.  I:On page 5 paragraph 2, the statement "we checked other systematic reviews to see similarities and differences in already published works" is suggesting that this is a systematic rather than scoping review.  S:You may want to remove "other". | Thank you for highlighting this, it has been amended and the word “other” has been removed for clarity on page 5. |
| Conflicting timelines for data retrieval.  I:On page 5 paragraph 3, you state that "Studies published before 2013 were excluded". However, you also state that "The data collected and published in the report spans from 2011-2021 and is the most recent and up to date evidence of the impact of race within the healthcare system".  S: Please clarify the correct date filter used. | Thank you for pointing this out. We have changed the timeline to include the date range of 2011-2013 on page 6 paragraph 1. |
| Crosscheck the content of Table 1.  I: On page 5 paragraph 2, you write that "Terms involving pregnancy AND health literacy AND BAME or ethnic minority group were combined for maximum results, see Table 1 for detailed search terms that were used" but on page 6 paragraph 1, you write that "Studies excluded during the full text screening stage were put into the categories described in Table 1". However, Table 1 is labelled "Table 1: Inclusion and Exclusion Criteria".  S: Please correct and label tables accordingly. Include the table on search terms. | Thank you for flagging. The error has been amended. The table including all search terms has been added to Appendix 1, which can be found on pages 31 and 32. |
| It is not clear how the conclusion that maternal health outcomes were not impacted by health literacy for BAME groups was made.  I: On page 10 paragraph 2 you state that "Although the studies generally advocate for increased health literacy in BAME groups, only one study by de Matta Machado Fernandes et al., described the impact that EBP and knowledge of healthcare had on health outcomes (18). More commonly, studies focused on the impact health literacy had on patient experience rather than health outcomes (28,32)". Also, on page 11 paragraph 1, you state "It is important to note that though the interventions found throughout the studies demonstrated an increase in health literacy within BAME populations, maternal health outcomes were not impacted".  S: Please check and correct for clarity. | Thank you for your feedback. We have discussed this and made the conclusion clearer within the discussion. This can be seen in the revised manuscript on page 20 in the first paragraph, “It is important to note that though the interventions found throughout the studies demonstrated an increase in health literacy within BAME populations, maternal health outcomes were minimally impacted.” Making it more clear to the reader that though health literacy was impacted, maternal health outcomes through evidence based practices were only seen to be improved in one of the 19 articles in the scoping review. |
| Present the presence or absence of key findings on the aim/objectives  I: On page paragraph, you reiterate that "This review aimed to evaluate the impact of health literacy on maternal health outcomes and patient experience among BAME pregnant women". However, these findings are not clearly presented.  S: Clearly identify the impact (or lack of impact or data) of health literacy. Were patient experiences and outcomes better or worse or not impacted or not reported in relation to the health literacy of the women? | Thank you for your feedback. We have amended our discussion to make the findings regarding the impact of health literacy more clear throughout the conclusion. This can be seen throughout the revised manuscript:  “Health inequalities that persist in BAME groups need to be mitigated through more research and investment into interventions. The impact of digital and community-based public health interventions was explored and found to be successful tools in increasing health literacy in BAME women. The global aspect of this review identified different interventions that aim to help BAME pregnant women that could be explored in other settings and provided recommendations for future research.” |
|  |  |
| Reviewer 2 Comments | Our Response |
| Abstract  • Main research question and key findings are summarised well in the abstract. | Thank you for your positive feedback. |
| Introduction  • Would be good to have some justification of why there is a focus on adverse health outcomes in pregnancy. | Thank you for your feedback, this note has been implemented through the manuscript as:  “The concept of health literacy is directly linked to socioeconomic status and thus connected to the social determinants of health... The international push towards decreasing maternal mortality is only possible if health inequalities are addressed and at risk BAME groups are given the proper attention during childbirth experiences.” |
| • First paragraph of page three – last sentence – “The concept of health literacy is directly linked to the social determinant of health and thus connected to socioeconomic status (6,7).” Should socioeconomic status be first? | Thank you for pointing this out; it is a helpful comment. We have addressed this by revising: "The concept of health literacy is directly linked to socioeconomic status and thus connected to the social determinant of health." This change aligns with the literature highlighting the intricate relationship between health literacy and socioeconomic factors, reinforcing the importance of considering social determinants in health outcomes. |
| • Second paragraph of page three – first sentence – why does maternal have a capital M? | Thank you for highlighting this oversight. We have corrected the term to “maternal” as suggested on page 3. We appreciate your attention to detail. |
| • Third paragraph of page three and second sentence – et al. is in italics. | Thank you for highlighting this error. We have corrected the term to “et al” as suggested on page 3. We appreciate your attention to detail. |
| • Best to replace Lang (reference 12) with a more recent reference if available or not include. | Thank you for pointing this out. We have discussed it internally and found a new source from 2024 that conveys the message we are emphasizing. Lang (reference 12) has now been replaced with Joseph et al, as seen on the reference list, located on page 26. This source highlights the fault of health care providers and lack of research on the increasing rise of maternal mortality in the United States. |
| • Good use of background info to justify research question. | Thank you for your positive feedback. Your constructive feedback reinforces the importance of this research. Your thoughtful review is truly valued. |
| Results • Page 7 – last line “Articles were only selected if they were published after 2013.” – can be removed as it is part of Methods. | Thank you for highlighting this error. We have removed the statement “Articles were only selected if they were published after 2013.” on page 10 as suggested and left it in the methods section on page 6. We appreciate your attention to detail. |
| • Page 8 – first line. Dash not needed. | Thank you for highlighting this error. We have taken away the dash on the bottom of page 8. |
| • Page 9 – penultimate sentence of third paragraph: “The intervention patient incorporated patient awareness of pregnancy through leaflets,…” Was the first use of “patient” used in error? | Thank you for highlighting this error. We have made the corrections and removed “patient” as suggested on page 17. We appreciate your attention to detail. |
| • Page 12 – first sentence of third paragraph doesn’t make sense: “For example, a study by Fernandez-Gutierrez et al. compiled a systematic review of health literacy frameworks aimed to help immigrants (40) interventions and themes yet needing more discussion on results.” | Thank you for highlighting this. The incorrect reference number has also been amended. It has been amended to “For example, a study by Fernandez-Gutierrez et al. compiled a systematic review of health literacy frameworks aimed to help immigrants (39). The interventions and themes were listed, but there was a need for more discussion on results and the communities that were impacted.” This amendment can be seen on page 20. |
| • There is reference to a table 3 but this does not appear to be included – should this refer to table 2? | Thank you for highlighting this error. We have rightly labelled it as Table 2 on page 10. We appreciate your attention to detail. |
